# The Potential Relationship Between HIF-1α and Amino Acid Metabolism After Hypoxic Ischemia and Dual Effects on Neurons

**DOI:** 10.3389/fnins.2021.676553

**Published:** 2021-08-18

**Authors:** Kexin Li, Yang Zheng, Xiaoming Wang

**Affiliations:** Department of Radiology, Shengjing Hospital of China Medical University, Shenyang, China

**Keywords:** hypoxic-ischemic injury, hypoxia inducible factor, amino acid metabolism, neurogenesis, neural plasticity

## Abstract

Hypoxia inducible factor (HIF) is one of the major transcription factors through which cells and tissues adapt to hypoxic-ischemic injury. However, the specific mechanism by which HIF regulates amino acid metabolism and its effect on neurons during hypoxic ischemia (HI) have remained unclear. This study analyzed the changes in cerebral metabolism of amino acids after HI by using ^1^H-MRS and investigated the relationship between the changes in cerebral metabolism of amino acids and HIF-1α as well as the potential effects on neurons. Newborn pigs were used as an HI model in this study. Twenty-eight newborn Yorkshire pigs (male, 1.0–1.5 kg) aged 3–5 days were selected and randomly divided into experimental groups tested at 0–2 h (*n* = 4), 2–6 h (*n* = 4), 6–12 h (*n* = 4), 12–24 h (*n* = 4), 24–48 h (*n* = 4), and 48–72 h (*n* = 4) after HI, and a control group (*n* = 4). After the modeling was completed, ^1^H-MRS imaging was conducted, followed by immunohistochemical staining of HIF-1α, NeuN, and doublecortin (DCX), and immunofluorescence of glutamic oxaloacetic transaminase (GOT)-1, GOT2, glutathione synthase (GS), glutamate-cysteine ligase catalytic subunit (GCLC), and glutamate-cysteine ligase modifier subunit (GCLM) in brain tissues. The expression of HIF-1α exhibited two increases after HI injury. The first time was opposite to the trends of change of GOT2, aspartic acid, and the number of neurons, while the second was consistent with these trends, suggesting that HIF-1α may have a two-way induction effect on neurons by regulating GOT2 after HI. HIF-1α was closely related to GCLM expression, and GSH level was correlated with the number of hippocampal neurons, indicating that HIF-1α may regulate GCLM to promote GSH synthesis and additionally play a neuroprotective role.

## Introduction

Neonatal hypoxic ischemic encephalopathy (HIE) refers to neonatal brain injury caused by perinatal asphyxia or decreased placental perfusion, the main pathogenesis of which is hypoxic ischemia (HI). HI can lead to energy failure and in turn triggers a cascade of injury and causes disorders of nervous system structure and function (Douglas-Escobar and Weiss, 2015; Millar et al., 2017). While the neonatal brain has a strong inherent capacity to recover, after HI injury, the nervous system can promote nerve recovery and reduce nerve injury through multiple pathways, including regulating signal transduction as well as neurotransmitter transmission, etc. (Ismail et al., 2017).

Hypoxia inducible factor (HIF) is a kind of transcription factor responsible for regulating oxygen homeostasis, which is composed of α and β subunits. After HI, HIF-1α promotes nerve recovery by regulating angiogenesis, neurogenesis, glucose transport, and metabolism (Chen et al., 2009; Vetrovoy and Rybnikova, 2019; Zhu et al., 2019). Under hypoxic conditions, prolyl 4-hydroxylase (PHD) activity is inhibited, which reduces the degradation of HIF-1α and increases HIF-1α expression, further promoting the transcription of HIF-1α target genes. This in turn boosts the expression of EPO and VEGF, among others, and facilitates angiogenesis (Chu and Jones, 2016; Yang et al., 2018). HIF-1α affects the differentiation and neurogenesis of neural stem cells by regulating Notch and Wnt/β-catenin signaling pathways (Chen et al., 2018), and it also reprograms cell metabolism to maintain cell energy homeostasis under hypoxia by upregulating many metabolic enzymes, including glucose and lactate transporters, and pyruvate dehydrogenase kinase (Parks et al., 2017; Kierans and Taylor, 2021; Paredes et al., 2021).

In recent years, the regulation of amino acid metabolism by HIF has gradually attracted increasing attention. It has been reported that HIF-1α promoted the synthesis of glutathione (GSH) in a bone regeneration model by upregulating glutaminase (GLS), reduced the damage of reactive oxygen species (ROS), thus promoting the survival of osteogenic cells, and further provided a new therapeutic strategy for stem cell therapy (Stegen et al., 2016). Besides, HIF-1α can contribute to the conversion of glutamine (Gln) to α-ketoglutarate (α-KG), which can be used in the glycolytic or tricarboxylic acid (TCA) cycle to generate energy (Jiang et al., 2017; Stegen et al., 2019).

After HI injury, complex change in the cerebral metabolism of amino acids occurs. It can affect the transmission of neurotransmitters and regulate the signal transduction of nerve cells (Blaise et al., 2017; Pregnolato et al., 2019), for example, the increase of glutamic oxaloacetic transaminase (GOT) after neonatal HI injury helps to reduce the excitotoxicity of glutamate (Glu) (Perez-Mato et al., 2016). The malate-aspartate shuttle (MAS) process includes amino acid conversion and is also coupled to the electron transport chain to facilitate energy homeostasis (Satrustegui and Bak, 2015; Beckervordersandforth, 2017). In addition, the increase of cerebral GSH after HI may play a neuroprotective role, the synthesis of which requires the participation of glutathione synthase (GS), glutamate-cysteine ligase catalytic subunit (GCLC) and glutamate-cysteine ligase modifier subunit (GCLM) (Lian et al., 2018; Li et al., 2021). However, it remains unclear the relationship between HIF-1α and the metabolism of amino acids after HI, and whether neuronal changes are due to the metabolism of amino acids by HIF-1α regulation. ^1^H-MRS imaging is a non-invasive strategy to determine the levels of cerebral metabolites, and it can be used to assess the changes in metabolites and the degree of injury after HI (Shibasaki et al., 2018; Dhamala et al., 2019). This study was established to analyze the changes in the metabolism of amino acids after HIE by using ^1^H-MRS and to investigate the relationship between the changes in the metabolism of amino acids and HIF as well as the potential effects of HIF on neurons.

## Materials and Methods

### Experimental Animals and HI Modeling

**FIGURE 1:**
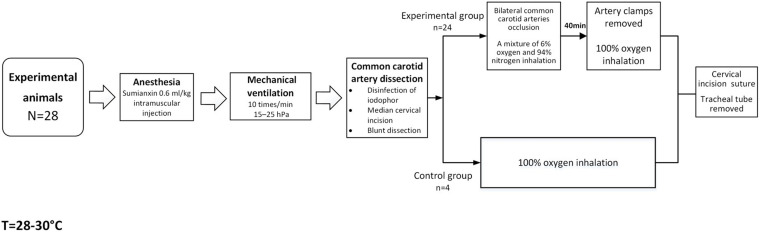
Flow chart of animal modeling.

Newborn pigs (*n* = 28) aged 3–5 days (bodyweight 1.0–1.5 kg, male) were used as experimental animals in this study. They were randomly divided into experimental groups, namely, 0–2 h (*n* = 4), 2–6 h (*n* = 4), 6–12 h (*n* = 4), 12–24 h (*n* = 4), 24–48 h (*n* = 4), and 48–72 h groups (*n* = 4), according to the different times after HI, and a control group (*n* = 4). The procedures to which the experimental animals were subjected were approved by the Animal Care and Use Institutional Committee of China Medical University (approval number 2015PS337K), and performed in accordance with the *Laboratory Animal Care and Use Guidelines* issued by the National Research Council. The modeling process is shown in Figure 1. The drugs and equipment used were as follows: sumianxin (Changchun Institute of Military Medical Research, Changchun, China) used in animal anesthesia; a mixture of 6% oxygen and 94% nitrogen (Dalian Special Gas, Dalian, China); U-25T bi-level airway pressure ventilator (BMC Medical Co., Ltd., Tianjin, China); and Heal Force pulse oximeter (Hexin Zondan Medical Equipment Co., Ltd., Shenzhen, China).

### ^1^H-MRS Scan and Data Postprocessing

An Achieva 3.0T magnetic resonance scanner (Philips Healthcare, Netherlands) was employed as the MR equipment, with a gradient coil transmission and 8-channel RF coil reception. A single-voxel PRESS sequence was used for ^1^H-MRS. The scanning parameters were as follows: TR = 2,000 ms, TE = 37 ms, NSA = 64, and VOI = 10 × 10 × 10 mm, with the basal ganglia being manually outlined as the region of interest (ROI) (Zheng and Wang, 2018). Shimming and fluid attenuation were conducted automatically by the scanner. LCModel software package was employed for further processing of the ^1^H-MRS results (metabolites included Glu at 2.04–2.35 ppm and 3.75 ppm, Gln at 2.35 ppm, Asp at 2.71 ppm, and GSH at 2.95 ppm; Moss et al., 2018).

### Immunohistochemistry and Immunofluorescence Staining

The brain tissue was fixed with formaldehyde and sliced into 4-mm-thick coronal sections incorporating the hippocampus, basal ganglia, and cortex. A Leica BOND-MAX^TM^ automatic staining machine (Leica, Germany) was used to conduct the immunohistochemical staining for HIF-1α (1:500; ab16066; Abcam), the neuronal marker NeuN (1:2,000; ab128886; Abcam), and neuronal migration protein doublecortin (DCX) (1:100; YP1091; Immunoway).

For GS, GCLC, GCLM, GOT1, and GOT2, immunofluorescent staining was performed. The staining steps were as follows: deparaffinization with xylene and alcohol hydration with gradient concentrations; antigen repair (0.01 M citrate buffer, pH 6.0, microwave for 37 min); blocking of endogenous peroxidase activity and non-specific antibody binding (3% hydrogen peroxide and normal goat serum, incubated at room temperature for 40 min); incubation with primary antibody overnight at 4°C (primary antibodies used: anti-GCLC antibody, 1:100, ab53179; anti-GCLM antibody, 1:100, ab154017; anti-GS antibody, 1:100, ab124811; anti-GOT1 antibody, 1:100, 60317-1-Ig; anti-GOT2 antibody, 1:100, 14800-1-AP); incubation with secondary antibody at room temperature for 4 h (secondary antibodies used: Alexa Fluor 488-labeled goat anti-mouse IgG 1:100, Immunoway, RS3208; Alexa Fluor 488-labeled goat anti-rabbit IgG 1:100, Immunoway, RS3211); and finally nuclear staining by incubation with 4,6-diamidino-2-phenylindole dihydrochloride (DAPI) for 5 min.

The immunohistochemistry images were collected using NIS-Elements F software (version 4.6; Nikon, Japan). The immunofluorescence images were collected using a confocal laser scanning microscope (LSM880; Zeiss, Göttingen, Germany) (×400). Three high-magnification (×400) fields from parietal cortical and hippocampal region were randomly and manually selected. Each image was analyzed automatically by ImageJ software (Java1.6.0; National Institutes of Health). The mean optical density (OD) of three fields was used to represent the staining intensity of the section.

### Statistical Analysis

One-way analysis of variance and LSD-*t-t*est were employed to analyze the differences between the subgroups, while Pearson’s correlation analysis was used to evaluate the correlations, with *P* < 0.05 being considered statistically significant. All statistical analyses were conducted by SPSS (version 22.0; IBM, Armonk, New York) and GraphPad Prism (version 8.0.2; GraphPad Software, San Diego, California).

## Results

### Changes in Asp, GSH, Glu, and Gln Content Observed by ^1^H-MRS After HI Injury

**FIGURE 2:**
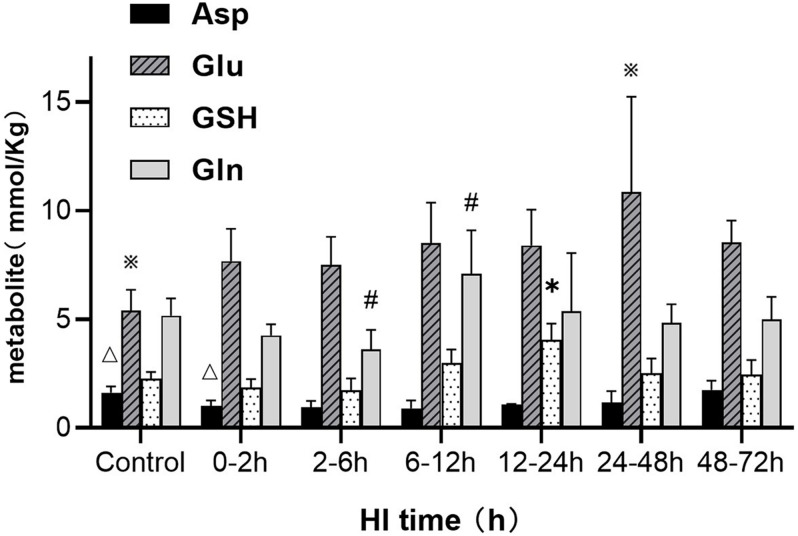
Changes in Asp, Glu, Gln, and GSH after HI injury (△, ※, *, # indicate a significant difference in a pairwise comparison, *P* < 0.05). The data are expressed as mean ± SD.

^1^H-MRS was used to observe the changes in the cerebral metabolism of amino acids at different time points after HI, as shown in Figure 2. Asp exhibited a tendency to initially decrease and then increase after HI; the Asp content at 0–2 h after HI was significantly reduced compared with that in the control group (*P* = 0.032, LSD-*t-t*est). GSH content at 12–24 h after HI was significantly higher than that in the other groups (*P* < 0.05, LSD-*t-t*est). Glu content at 24–48 h after HI was significantly higher than that in the control group (*P* = 0.002, LSD-*t-t*est). Finally, Gln content initially decreased and then significantly increased at 6–12 h after HI (*P* < 0.05, LSD-*t-t*est).

### Changes in GOT1, GOT2, GS, GCLC, and GCLM Expression After HI Injury

**FIGURE 3:**
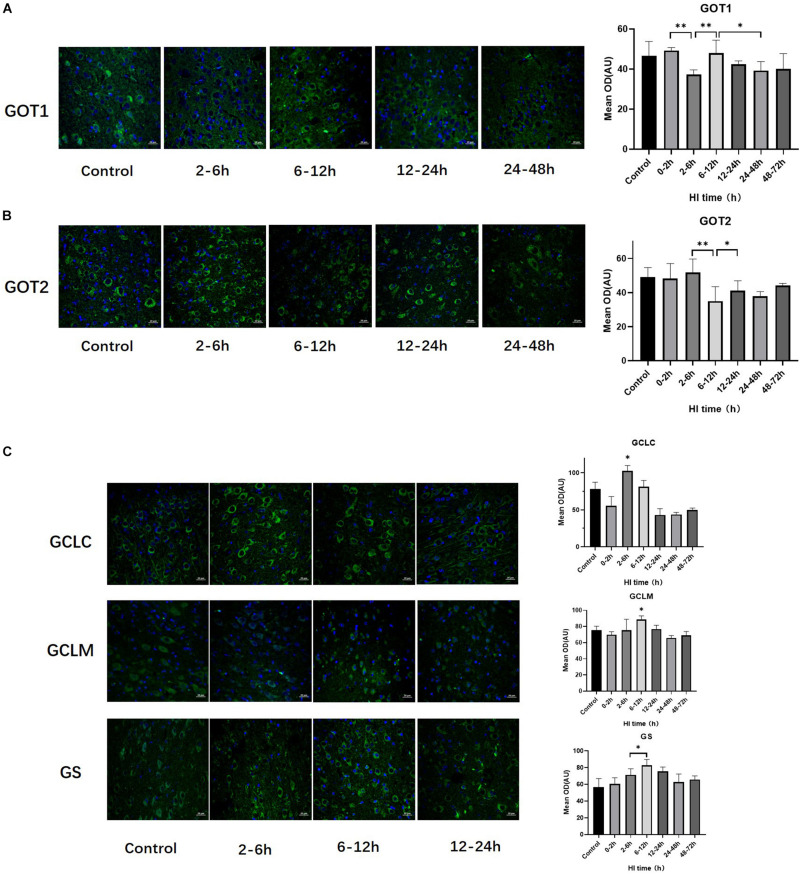
Changes in the expression of GOT1, GOT2, GCLC, GCLM, and GS after HI injury (×400). **(A)** Tendency chart for cerebral GOT1 staining and mean optical density in the control group and HI groups at 2–6, 6–12, 12–24, and 24–48 h after HI. Green fluorescence represents GOT1. The fluorescence intensity of GOT1 decreases at 2–6 h, then increases at 6–12 h, and significantly decreases again at 24–48 h after HI. **(B)** Tendency chart for cerebral GOT2 staining and mean optical density in the control group and HI groups at 2–6, 6–12, 12–24, and 24–48 h after HI. Green fluorescence represents GOT2. The fluorescence intensity of GOT2 decreases at 6–12 h and then increases at 12–24 h after HI. **(C)** Tendency chart for the cerebral GCLC, GCLM, and GS staining, and mean optical density in the control group and the groups at 2–6, 6–12, and 12–24 h after HI. Green fluorescence represents GCLC, GCLM, and GS. The fluorescence intensity of GCLC, GCLM, and GS peaks at 2–6 h and 6–12 h after HI. ^∗∗^*P* < 0.01, ^∗^*P* < 0.05; data are expressed as mean ± SD.

The trends of change of GOT1, GOT2, GS, GCLC, and GCLM expression over time after HI injury are shown in Figure 3. GOT1 and GOT2 were significantly decreased at 2–6 h and 6–12 h after HI (both *P* < 0.01, LSD-*t-t*est), and significantly increased at 6–12 h and 12–24 h after HI, respectively (*P* < 0.01, *P* < 0.05, LSD-*t*-test). Peaks in the expression of GCLC and GCLM occurred at 2–6 h and 6–12 h after HI, respectively, and such expression was significantly higher than that in the other groups (*P* < 0.01, *P* < 0.05, LSD-*t-t*est). There was a tendency for an initial increase and then a decrease in GS expression after HI injury, which significantly increased at 6–12 h (*P* = 0.047, LSD-*t*-test).

### HIF-1α Expression in Brain Tissue After HI Injury and Its Relationship With the Expression of GS, GCLC, GCLM, GOT1, and GOT2

**FIGURE 4:**
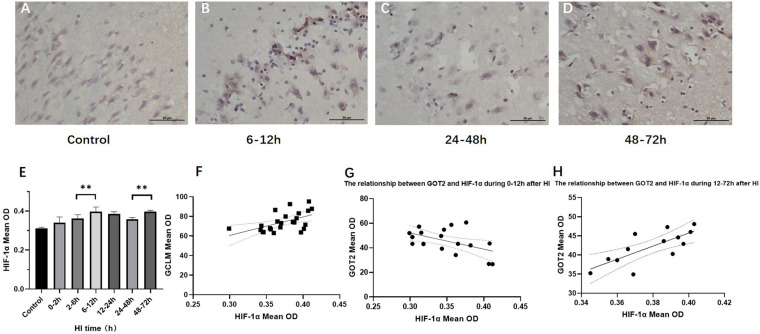
Changes in HIF-1α expression (×400) and scatter plot of correlation with GCLM and GOT2 after HI. **(A–D)** Immunohistochemical staining for HIF-1α in the control group and HI groups at 6–12, 24–48, and 48–72 h after HI. **(E)** HIF-1α expression level gradually increases after HI, reaching the first peak in the 6–12 h group, significantly decreases in the 24–48 h group, and increases again in the 48–72 h group. **(F)** Scatter plot of the correlation between HIF-1α and GCLM expression. There is a significant positive correlation. **(G,H)** Scatter plot of the correlation between HIF-1α and GOT2 expression. In the control group and HI groups at 0–2, 2–6, and 6–12 h after HI, HIF-1α expression and GOT2 expression show a significant negative correlation. In the groups at 12–24, 24–48, and 48–72 h after HI, they instead show a significant positive correlation. ^∗∗^*P* < 0.01; data are expressed as mean ± SD.

Figure 4 shows the changes in HIF-1α expression in brain tissue over time after HI injury. There were two peaks in the HIF-1α expression level after HI, namely, at 6 − 12 h and 48–72 h (*P* < 0.01, LSD-*t-t*est). In the HI group, the HIF-1α expression level was significantly positively correlated with the GCLM expression level (*r* = 0.537, *P* = 0.007), while it was not significantly correlated with GCLC and GS (*P* = 0.917 and 0.055, respectively). The HIF-1α expression level was significantly negatively correlated with the GOT2 expression level in control group and at 0–12 h after HI (*r* = −0.542, *P* = 0.037), which reversed to a significant positive correlation at 12–72 h after HI (*r* = 0.762, *P* = 0.004). There was no significant correlation between the expression levels of HIF-1α and GOT1 (*P* = 0.549).

### Changes in the Numbers of Hippocampal and Cortical Neurons After HI Injury

**FIGURE 5:**
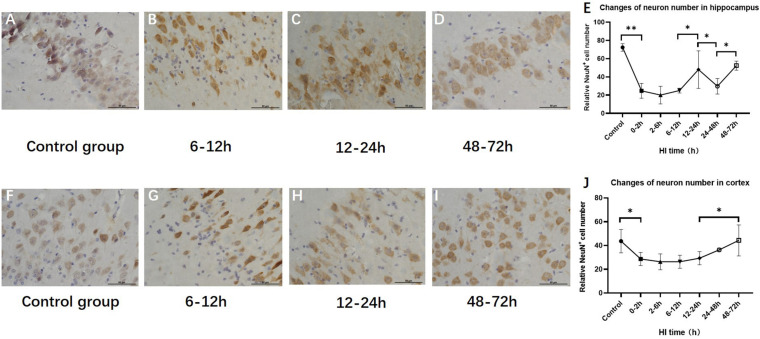
Changes in the number of NeuN-positive cells in the hippocampus and cortex after HI (×400). **(A)** NeuN immunohistochemical staining of the hippocampus in the control group. **(B–D)** NeuN immunohistochemical staining of the hippocampus in HI groups at 6–12, 12–24, and 48–72 h after HI. **(E)** The number of NeuN-positive cells in the hippocampus decreases at 0–12 h and significantly increases at 12–24 h after HI injury. **(F)** NeuN immunohistochemical staining of the cortex in the control group. **(G–I)** NeuN immunohistochemical staining of the cortex in groups at 6–12, 12–24, and 48–72 h after HI. **(J)** Compared with the control group, there is a significant decrease in the number of NeuN-positive cells in the cortex at 0–2 h after HI injury, and the number of NeuN-positive cells at 48–72 h after HI is significantly increased. ^∗∗^*P* < 0.01, ^∗^*P* < 0.05; data are expressed as mean ± SD.

The numbers of hippocampal and cortical neurons exhibited significant differences compared with those in the control group (*P* < 0.001 and *P* = 0.034, ANOVA). The numbers of hippocampal and cortical neurons both initially decreased and then increased. The number of neurons increased at 12–24 h (*P* < 0.01, LSD-*t-t*est) and 48–72 h (*P* < 0.01, LSD-*t-t*est), respectively (Figure 5 and Supplementary Figure).

### Changes in DCX Expression After HI Injury

**FIGURE 6:**
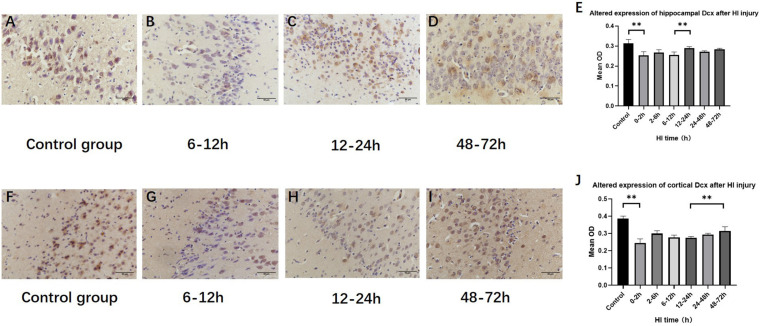
Changes in DCX expression in the hippocampus and cortex after HI (×400). **(A)** DCX immunohistochemical staining of the hippocampus in the control group. **(B–D)** DCX immunohistochemical staining of the hippocampus in HI groups at 6–12, 12–24, and 48–72 h after HI. **(E)** The expression of DCX in the hippocampus decreases at 0–2 h and significantly increases at 12–24 h after HI injury. **(F)** DCX immunohistochemical staining of the cortex in the control group. **(G–I)** DCX immunohistochemical staining of the cortex in groups at 6–12, 12–24, and 48–72 h after HI. **(J)** Compared with the control group, DCX expression significant decrease in the cortex at 0–2 h after HI injury, and the expression of DCX at 48–72 h after HI is significantly increased. ^∗∗^*P* < 0.01; data are expressed as mean ± SD.

The expression of hippocampal and cortical DCX exhibited significant differences among the HI groups and the control group (*P* < 0.001, respectively, ANOVA). The expression of hippocampal DCX decreased at 0–2 h (*P* < 0.01, LSD-*t-t*est) and increased at 12–24 h (*P* < 0.01, LSD-*t-t*est), while the expression of cortical DCX increased at 48–72 h (*P* < 0.01, LSD-*t-t*est) (Figure 6 and Supplementary Figure).

## Discussion

Hypoxia is a trigger of many pathological conditions such as HIE, which is usually accompanied by insufficient synthesis or increased consumption of glucose and amino acids. HIF is one of the major transcription factors through which cells and tissues adapt to hypoxia. By participating in the regulation of metabolic reprogramming, HIF can meet the body’s biosynthetic and energy requirements under hypoxic conditions (Semenza, 2012; Chen and Sang, 2016). Research performed to date on the mechanism by which HIF regulates cell metabolism has mainly focused on glucose and fatty acid metabolism (Bouthelier and Aragones, 2020), while the specific mechanism by which amino acid metabolism is regulated remains to be studied. In addition, owing to a large number of downstream target genes of HIF and its extensive effects, HIF-1α after HI injury can activate not only neuroprotection but also the signal pathways after HI injury (e.g., apoptosis), resulting in a dual effect of neuroprotection and nerve injury (Chen et al., 2007, 2009). In this study, we used ^1^H-MRS imaging to observe the dynamic changes of cerebral metabolites after HI injury and analyzed their correlations with HIF-1α expression and changes in the number of neurons to investigate the mechanisms by which HIF-1α regulates amino acid metabolism and neurons after HI injury.

### Correlation Between GSH Synthesis and HIF-1α Expression After HI Injury and the Effect of HIF-1α on Neurons

The neural synthesis of GSH involves the Glu, cysteine, and glycine as precursors. Glu is mainly derived from the transport by excitatory amino acid carrier 1 and the Glu-Gln shuttle (Aoyama et al., 2008; Sedlak et al., 2019). The first synthesis step of GSH is the formation of γ-glutamylcysteine (γ-GC), which is catalyzed by GCL, a heterodimer composed of GCLC and GCLM. GCLC acts as the catalyst, while the synthesis process modified and regulated by GCLM. In the second step, GSH is synthesized in a manner catalyzed by GS (Chen et al., 2005; Lian et al., 2018).

The ^1^H-MRS results in this study showed that Gln increased at 6–12 h and decreased rapidly at 12–24 h after HI, at which time the GSH content increased significantly. The immunofluorescence staining results of synthetases showed that GCLC reached its peak at 2–6 h after HI injury, and GCLM and GS reached their peaks at 6–12 h after HI, indicating that the synthesis of GSH was activated after HI injury. However, this study found that the changes of GCLC and GCLM were not synchronized, and the GCLC expression increased earlier than that of GCLM, suggesting that GCLC may have catalyzed the whole reaction. The 5′end of GCLM mRNA has an HIF-1α binding site, which can be used to induce GCLM expression by HIF-1α (Lu et al., 2015; Stegen et al., 2016). This study found that the HIF-1α expression was significantly positively correlated with the GCLM expression, but not significantly correlated with the GCLC and GS expression after HI injury, indicating that HIF-1α mainly promotes GSH synthesis by upregulating the GCLM expression after HI.

GSH is the most abundant antioxidant in cells, the increased content of which in cells can further reduce ROS production and alleviate oxidative stress injury (Moss et al., 2018). The decreased GSH content can enhance the cytotoxicity induced by glutamate or NO, resulting in neuronal injury (Ibi et al., 1999). Previous assessment of neuronal damage after HI by our team found that the main pathological change of neurons at 0–24 h was edema, which was a kind of mild reversible injury (Zheng and Wang, 2018; Dang and Wang, 2020). In this study, histopathological analysis confirmed that the number of hippocampal NeuN-positive cells initially decreased, which may due to neural antigen injury and diminishing the ability to bind anti-NeuN antibodies (Gusel’Nikova and Korzhevskiy, 2015). Then the number of the hippocampal NeuN-positive cells rebounded at 12–24 h after HI, consistent with the expression of DCX and the peak time of GSH, which probably means neurogenesis and neuroprotective effect of GSH in this period. GSH content decreased at 24–48 h after HI, at which time the number of neurons decreased again, suggesting that the changes of GSH after HI can reflect neuronal injury and recovery to a certain extent.

### The Relationship Between HIF-1α and Asp Metabolism After HI Injury and the Effect of HIF-1α on Neurons

Asp functions in regulating neurometabolism and biosynthesis; the MAS is one of the ways in which Asp participates in metabolic regulation. GOT is an important part of the MAS, which includes two isoenzymes: GOT1 and GOT2. The former is mainly present in the cytoplasm and is used to transform Asp and α-KG into oxaloacetic acid (OAA) and Glu in normal cells. The latter is present in the mitochondrial matrix and is employed to generate Asp and α-KG (Birsoy et al., 2015), which can transform Glu into α-KG to form part of the TCA cycle, and finally replenish the lost energy and reduce the nerve injury caused by Glu during ischemic stroke (Rink et al., 2017). Previous studies found that the electronic transport chain (ETC) is inhibited after hypoxia, Asp production is decreased (Garcia-Bermudez et al., 2018), and HIF-1α inhibits Asp metabolism by downregulating GOT expression (Melendez-Rodriguez et al., 2019; Bouthelier and Aragones, 2020). This study analyzed the relationships between HIF-1α, GOT, and Asp after HI, with the results showing that there were two peaks of HIF-1α expression after HI. The HIF-1α expression gradually increased at 0–12 h after HI, and reached its first peak at 6–12 h, while GOT2 expression decreased. There was a significant negative correlation between HIF-1α and GOT2 expression, and the Asp content also showed a downward trend during this period. At 12–72 h after HI, there was a significant positive correlation between HIF-1α and GOT2. The second peak of HIF-1α appeared at 48–72 h after HI, the GOT2 expression increased, as did the Asp content. This indicates that HIF-1α may affect Asp production by regulating GOT2 expression, and that HIF-1α has various effects during different recovery periods after HI. Previous studies also confirmed the “biphasic activation” phenomenon of HIF-1α after cerebral ischemia, which may cause dual effects of protection of and damage to neurons (Baranova et al., 2007; Arumugam et al., 2018). However, the specific mechanism by which HIF-1α regulates GOT and whether there is synergistic activation of multiple transcription factors remain to be studied (Chen and Sang, 2016).

In addition, this study found that HIF-1α had no significant correlation with GOT1, and the changes of GOT1 and GOT2 were not synchronized, which may be related to the difference in regulation between them. After ETC inhibition, GOT1 can catalyze the reductive carboxylation pathway of Gln alone to produce Asp, but this cannot completely compensate for the loss of Asp synthesis in mitochondria (Fendt et al., 2013; Perez-Mato et al., 2014; Birsoy et al., 2015). This study found that Gln increased significantly at 6–12 h after HI, GOT1 expression increased, and Asp showed an upward trend, suggesting that the Gln-dependent Asp synthesis may be one of the reasons for the increase in Asp content. However, further experiments are needed to clarify the degree of ETC inhibition after HI injury and the dynamic changes in intermediate metabolites.

The MAS plays an important role in the electron transfer process of the neuronal oxidative transport chain, and is involved in maintaining neuronal metabolism and synaptic function after cerebral ischemia (Juaristi et al., 2019; Xu et al., 2020). This study analyzed the effects of Asp metabolism and MAS changes on the number of neurons using histopathology. The results showed that there was an upward trend in the numbers of hippocampal and cortical neurons at 12–24 h after HI. At this time, GOT2 expression increased and GOT1 expression decreased, indicating the increased production of Asp and α-KG in the mitochondrial matrix, which can provide energy for neurons and reduce Glu excitotoxicity. Asp is the main raw material for pyrimidine synthesis (Lane and Fan, 2015) and participates in amino acid metabolism during cell proliferation. This study found that Asp, DCX and cortical neurons shared the same trends of change, suggesting that Asp may provide synthetic precursors during the neurogenesis, migration and recovery process of cortical neurons.

## Conclusion

The expression of HIF-1α exhibits two increases after HI injury. The first time is opposite to the trends of change of GOT2, Asp, and the number of neurons, while the second is consistent with these trends, suggesting that HIF-1α may have a two-way induction effect on neurons by regulating GOT2 after HI. HIF-1α is closely related to GCLM expression, and the GSH level was correlated with the number of hippocampal neurons, suggesting that HIF-1α may regulate GCLM to promote GSH synthesis and further play a neuroprotective role.

## Data Availability Statement

The raw data supporting the conclusions of this article will be made available by the authors, without undue reservation.

## Ethics Statement

The animal study was reviewed and approved by Animal Care and Use Institutional Committee of Shengjing Hospital affiliated by China Medical University.

## Author Contributions

KL: investigation, data curation, and writing-original draft. YZ: validation, resources, and supervision. XW: conceptualization, methodology, writing-review and editing, and project administration. All authors participated sufficiently to take public responsibility for its content, read and approved the submitted version.

## Conflict of Interest

The authors declare that the research was conducted in the absence of any commercial or financial relationships that could be construed as a potential conflict of interest.

## Publisher’s Note

All claims expressed in this article are solely those of the authors and do not necessarily represent those of their affiliated organizations, or those of the publisher, the editors and the reviewers. Any product that may be evaluated in this article, or claim that may be made by its manufacturer, is not guaranteed or endorsed by the publisher.

## Abbreviations

α-KG: α-ketoglutarate
γ-GC: γ-glutamylcysteine
Asp: aspartic acid
DCX: doublecortin
ETC: electronic transport chain
GCLC: glutamate-cysteine ligase catalytic subunit
GCLM: glutamate-cysteine ligase modifier subunit
Gln: glutamine
GLS: glutaminase
Glu: glutamate
GOT: glutamic oxaloacetic transaminase
GS: glutathione synthase
GSH: glutathione
HI: hypoxic ischemia
HIE: hypoxic-ischemic encephalopathy
HIF: hypoxia inducible factor
MAS: malate-aspartate shuttle
OAA: oxaloacetic acid
OD: optical density
PHD: prolyl 4-hydroxylase
ROS: reactive oxygen species
TCA: glycolytic or tricarboxylic acid.
