# Supplementary material for: The Potential Relationship Between HIF-1α and Amino Acid Metabolism After Hypoxic Ischemia and Dual Effects on Neurons
Source: Front Neurosci. 2021 Aug 18;15:676553. doi: 10.3389/fnins.2021.676553 (PMC8416424; doi:10.3389/fnins.2021.676553)

Supplementary figure (NeuN-×200)

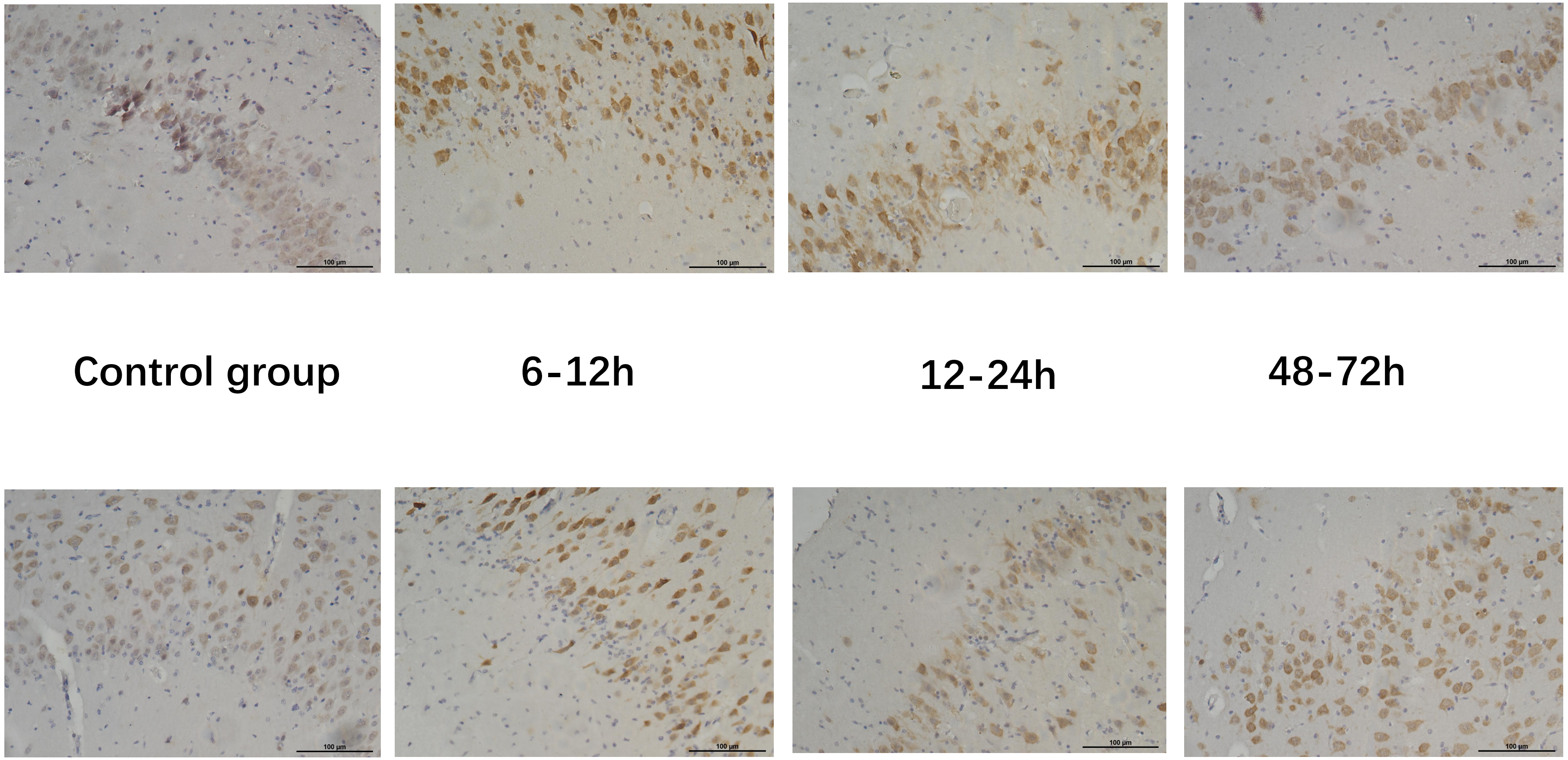

Hippo-
campus

Control group | 6-12h | 12-24h | 48-72h

Cortex

Control group | 6-12h | 12-24h | 48-72h

Supplementary figure (Dcx-×200)

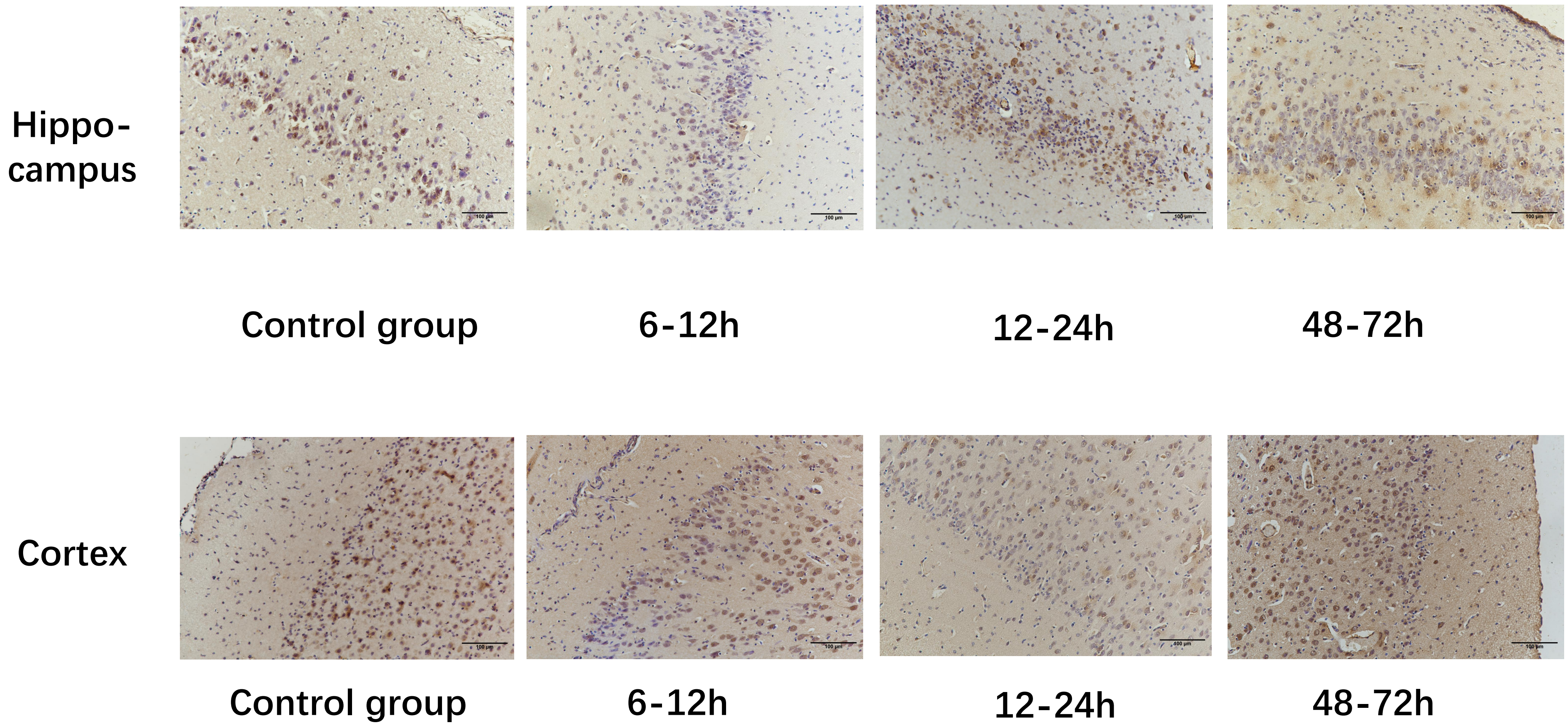

Supplement: Supplementary file 1 [file Data_Sheet_1.PDF]
